# Clinical course of untreated cerebral cavernous malformations: individual patient data meta-analysis

**DOI:** 10.1186/1745-6215-14-S1-P118

**Published:** 2013-11-29

**Authors:** Margaret Horne, Kelly Flemming, I-Chang Su, Christian Stapf, Robert Brown, Teresa Christianson, Ronit Agid, Karel terBrugge, Robert Willinsky, Susanne Maxwell, Gordon Murray, Rustam Al-Shahi Salman

**Affiliations:** 1 University of Edinburgh, Edinburgh, UK; 2 Mayo Clinic, Rochester, MN, USA; 3 Toronto Western Hospital, Toronto, Ontario, Canada; 4 Hôpital Lariboisière, Paris, France

## Background

The clinical course of untreated cerebral cavernous malformations (CCM) remains uncertain, partly due to small sample sizes and the infrequency of outcome events in previous studies. The dilemma about whether, when and how to treat patients would be informed by a more precise estimation of clinical course, identification of prognostic factors and derivation of prognostic models.

## Method

From a systematic review, we identified three prospective or retrospective hospital-based cohorts (Mayo Clinic, Rochester, MN; Toronto Western Hospital, Toronto; and Hôpital Lariboisière, Paris) and one prospective population-based cohort (Scotland) that could provide detailed data regarding clinical outcome between diagnosis and CCM treatment or last follow-up in adults with CCM. We will describe baseline characteristics and use survival analysis to calculate the risks of outcomes at specific times and identify their predictors using adjusted hazard ratios. We will pool the estimates from each study in meta-analyses using random-effects models, and quantify and investigate any heterogeneity between studies.

## Results

In three cohorts providing data to date (*n* = 745), between 29% and 46% of patients presented incidentally, 20-29% with seizure, 13-34% with haemorrhage, and 11-17% with focal neurological deficit. In follow-up these cohorts identified 105 symptomatic events, including 46 haemorrhages.

## Discussion

A large multinational meta-analysis of CCMs has been initiated. The inclusion of cohorts and their detailed data will be finalised by August 2013, with more than 1,000 patients to be included. We will present our initial results in November 2013. Our findings will help design future studies of the effects of treatment.

